# Multi-domain computerized cognitive training for children with intellectual developmental disorder: A randomized controlled trial

**DOI:** 10.3389/fpsyg.2022.1059889

**Published:** 2023-01-09

**Authors:** Jingsong Wu, Juan Peng, Zhaoying Li, Haiyin Deng, Zhenming Huang, Youze He, Jingnan Tu, Lei Cao, Jia Huang

**Affiliations:** College of Rehabilitation Medicine, Fujian University of Traditional Chinese Medicine, Fuzhou, Fujian Province, China

**Keywords:** computerized cognitive training, intellectual function, adaptive behavior, intellectual developmental disorder, children rehabilitation

## Abstract

**Purpose:**

To verify the effects of multi-domain computerized cognitive training on intellectual function and adaptive functioning in children with intellectual developmental disorder (IDD).

**Methods:**

Children with IDD were randomized to a multi-domain computerized cognitive training (CCT) group (n = 30) and control group (n = 30). Both groups received a 5-week training program. Intellectual function was assessed by Chinese-Wechsler Young Children scale (C-WYCSI) and adaptive functioning was assessed by the Chinese Vineland Adaptive Behavior Rating Scale (VABS-C), which were used at baseline, post-training, and 3-month follow-up.

**Results:**

There were significant differences for intellectual function and adaptive functioning between the two groups. The CCT group showed significant improvements in total full-scale intelligence quotient (FSIQ) score the Wechsler Intelligence Scale (*F*[60] = 31.97, *p* < 0.01) and its subdomain VIQ score (*F*[60] = 33.83, *p* < 0.01). For adaptive functioning, CCT had a better adaptive developmental quotient (ADQ) score (*F*[60] = 28.05, *p* < 0.01), and subdomain communication (*F*[60] = 10.86, *p* < 0.01) and socialization scores (*F*[60] = 4.35, *p* < 0.015). Moreover, there was a positive correlation between FSIQ changes and ADQ changes in the CCT group (r_s_ = 0.74, *p* < 0.01). A greater increase in VIQ score was associated with a greater increase in adaptive functioning (bootstrapping CI: [0.16, 3.30]) in the CCT group.

**Conclusion:**

Multi-domain CCT improves the intellectual function and adaptive functioning of children with IDD.

## 1. Introduction

Intellectual developmental disorder (IDD) is a neurodevelopmental disorder characterized by substantial limitations in intellectual function and adaptive functioning (American Psychiatric Association and American Psychiatric Association, 2013), with a worldwide prevalence of approximately 1%–3% (McKenzie et al., 2016). Intelligence measures general cognitive functions (Deary, 2012), including visual perception (Wan et al., 2017; Cui et al., 2019), attention (Kirk et al., 2016; Kirk H. E. et al., 2017), memory (Söderqvist et al., 2012; Purugganan, 2018), and reasoning (Hilger et al., 2017; Ma et al., 2017). Impairment of intellectual function implies impairment of cognitive domains (Yang et al., 2020), which may result in poor participation in daily activities, learning disabilities, and social difficulties in children, and restriction of overall development (Kirk et al., 2016; Schwartz et al., 2016; Ke et al., 2020).

In recent years, computerized cognitive training (CCT) has gradually emerged as a new treatment technology to improve neurodevelopmental disorders (Chen et al., 2013). Numerous studies have shown that CCT is effective for IDD (Söderqvist et al., 2012; Henrik et al., 2015; Kirk et al., 2016; Kirk H. E. et al., 2017). Studies have also shown that children with IDD prefer computer technology in leisure and learning activities (Hartin et al., 2016). Using CCT could target impaired cognitive domains by providing timely feedback from multi-sensory channels such as those of sight, hearing, and touch, which could improve the performance, learning initiative, and motivation of these children (Serret et al., 2014; Zhang et al., 2017; Denervaud et al., 2020). Furthermore, studies have proven that cognitive functions, such as attention, can be positively influenced *via* targeted training (Henrik et al., 2015; Kirk et al., 2016). However, these gains are generally restricted by the training domain, and the generalization effect is limited due to the untrained tasks (Cui et al., 2019b).

Multi-domain cognitive training may be more helpful to children with developmental disorders (Kirk H. et al., 2017). According to the characteristics of cognitive impairment in children with IDD, designing a comprehensive multi-domain CCT training program may be more conducive to the recovery of children with IDD. Moreover, studies have shown a strong link between cognitive function and adaptive behavior (Tassé et al., 2016; Cordeiro et al., 2020), which means that multi-domain CCT may also be beneficial to improve adaptive functioning.

We aimed to verify the effect of multi-domain CCT on intellectual function and adaptive functioning in children with IDD. We hypothesized that multi-domain computer-assisted cognitive training could enhance the comprehensive cognitive function of children with IDD and, thereby, improve their intellectual performance and adaptive functioning.

## 2. Materials and methods

### 2.1. Study design

We conducted a single-blinded randomized controlled trial, whereby clinical assessments were administered to all participants at pre-intervention, post-intervention (5 weeks), and at follow-up (3 months after post-intervention). The protocol for this trial was approved by the Ethics Committee of the Rehabilitation Hospital Affiliated to Fujian University of Traditional Chinese Medicine (Approved number: 2019KY-004-01) in 2019. This study was performed in accordance with the guideline of the Declaration of Helsinki, and informed consent was obtained from all participants and their legal guardians before screening. The details of this trial were also registered in the China Clinical Trial Registry (Registration number: ChiCTR1900024413; Registration date: 10/07/2019).

### 2.2. Participants

Eligible participants were recruited from special education schools, child rehabilitation training centers, and local nonprofit organizations in Fuzhou City, China. We included children aged 4–6.5 years who met the DSM-5 IDD criteria as diagnosed by clinicians, as well as children with a the full-scale intelligence quotient (FSIQ) score < 70, as measured by the Chinese-Wechsler Young Children Scale of Intelligence (C-WYCSI; Gong Yaoxian, 1992); and an adaptive developmental quotient (ADQ) < 70, as measured by the Vineland Adaptive Behavior Scale-Chinese Version (VABS-C; Yao and Gong, 1991). We excluded children with any visual, auditory, or motor impairment to the extent that it was impossible to complete the assessment and training; those who failed to complete the assessments and training due to the inability to follow instructions or poor cooperation; and those who participated in any activity within the past 30 days that may have affected the results of this study, such as performing the same test or participating in another clinical research trial.

### 2.3. Intervention

All therapists were licensed rehabilitation therapists with at least 3 years of clinical experience in the treatment of children with IDD, and all training sessions were based on intervention manuals and arranged according to the individual characteristics of each child. The therapists for the CCT group were only responsible for switching the machine on and off and monitoring the treatment status of the children, and did not participate in the cognitive training process. Therapists for the control group carried out one-to-one training according to the intervention. The interventions were conducted in quiet treatment rooms.

During the intervention period, each child received five training sessions, five times a week, for 20 min each time. Training for both groups covered four cognitive domains, namely visual perception, attention, memory, and reasoning, but the medium and content of training were different. The CCT group was trained through an electronic platform, while the control group was trained by a therapist.

#### 2.3.1. CCT experimental group

**Figure 1:**
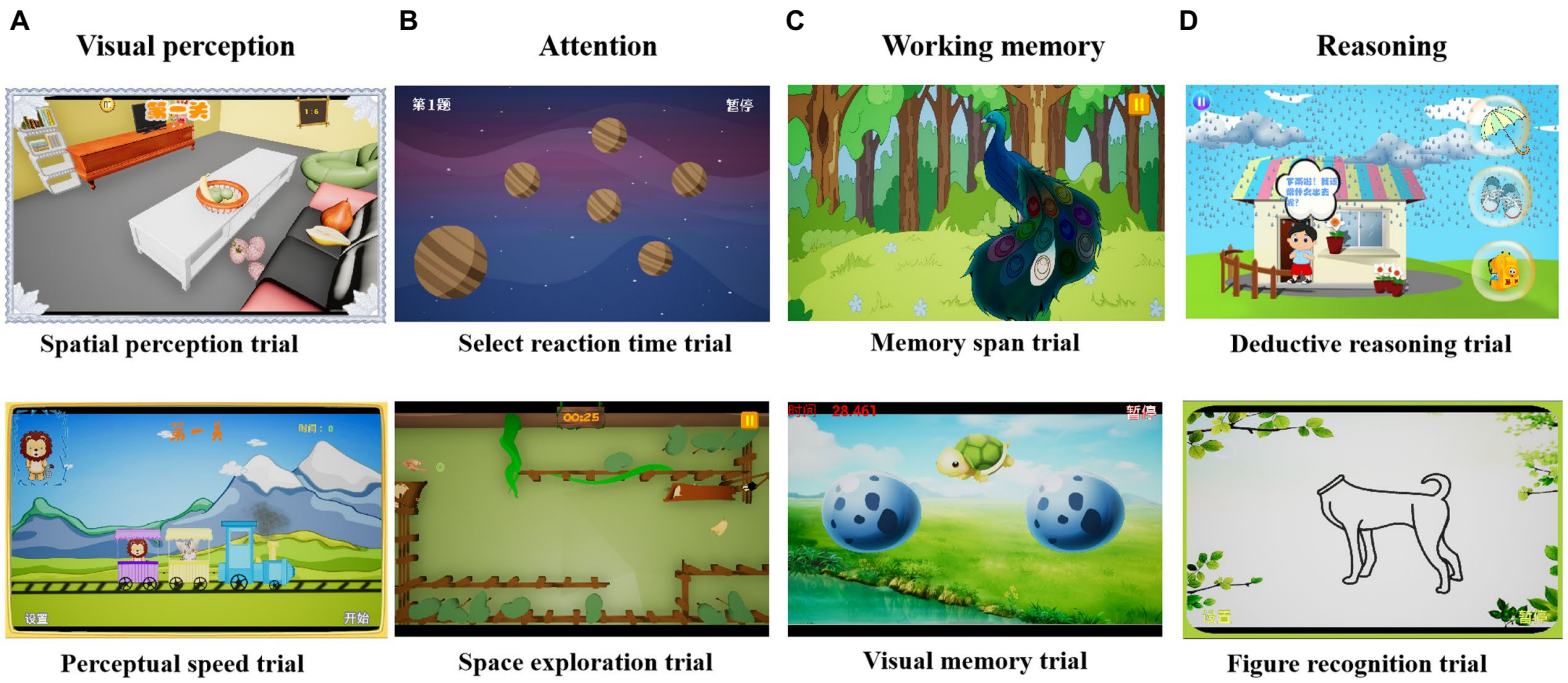
The eight trials of computerized cognitive training. **(A)** Visual perception block, including the spatial perception and perceptual speed trials. **(B)** Attention block, including the select reaction time trial and space exploration trial. **(C)** Working memory block, including the memory span trial and visual memory trial. **(D)** Reasoning block, including the deductive reasoning and figure recognition trials.

The children performed training sessions on electronic tablets. The training program task consisted of four blocks (visual perception, attention, memory, and reasoning) and each included two trials (eight trials in total) which are most relevant to the cognitive impairment of children with IDD (Figure 1). Each trial had three levels and was adaptive: the computer-assisted programs provided immediate feedback according to the child’s performance and adjusted the difficulty of the exercises and reinforcement methods. When a child reached 80% correct answers, the difficulty of the training increased. In the training process, the embedded human-computer interaction function of the system provided visual and verbal guidance. Participants received encouragement and feedback after completion of each level, such as stickers and fireworks/thumbs up on the screen as the task reward. During each training session, four of the eight trials were randomly selected. Each session is 20 min long, each trial lasted for 5 min, for a total training time of 20 min. The trials were presented as follows.

##### 2.3.1.1. Perceptual speed trial (visual perception)

The children help a ticket inspector choose the corresponding small animal ticket. There are several animals on an advancing train and, according to the animals randomly appearing in the upper left corner of the screen, the children click on the matching animals to make them enter the train.

##### 2.3.1.2. Spatial perception trial (visual perception)

This task aims to classify similar objects in the room and then put them back where they should be stored, such as vegetables, fruits, and electrical appliances. These are all common items in the children’s daily lives.

##### 2.3.1.3. Select reaction time trial (attention)

The task module interface is a randomly changing color of asteroids revolving around a large planet. The children need to compare the randomly appearing and changing color of asteroids to determine consistency with the color of the giant planet and make a quick response.

##### 2.3.1.4. Space exploration trial (attention)

A monkey is in a maze and wants to eat a banana. The children need to analyze the complicated topography and, using as few attempts and steps as possible, help the monkey cross the maze to successfully eat the banana.

##### 2.3.1.5. Memory span trial (working memory)

The task module interface involves a peacock which has 20 large feathers and is looking for friends. The distance between the peacock and the friend starts as five steps. Each time the peacock illuminates a feather, the children need to remember the corresponding feather position and respond correctly. Each time the selection is correct, the distance between the peacock and the friend reduces by one step. The position of the feathers is entirely random.

##### 2.3.1.6. Visual memory trial (working memory)

A group of small animals is hidden in the eggshell. The children need to memorize the location of the small animals and, when the small animals are hidden, find the position according to the instruction picture provided by the training module. The location of the animal matches the picture.

##### 2.3.1.7. Deductive reasoning trial (reasoning)

The children are required to propose solutions to dilemmas encountered in the project environment based on their daily life experiences. These may include having to go out on rainy days and what items should be taken with.

##### 2.3.1.8. Figure recognition trial (reasoning)

A random picture with a crucial missing part will appear on the screen. The children need to indicate the missing part according to common sense and after observation.

#### 2.3.2. Traditional cognitive training (control group)

The children in the control group underwent training administered by the traditional cognitive training program concerning the contents of rehabilitation for IDD in the 2016 Practical Child Rehabilitation Medicine (2nd ed; Li, 2016). All therapists were trained before the trial, and the four control trials involved visual perception, attention, memory, and reasoning ability. The therapist trained each child using rehabilitation teaching aids (such as building blocks, beads, and cards), and judged and adjusted the difficulty of the training based on the child’s performance. If the correct rate of training exceeded 80%, the therapist adjusted the difficulty. Each trial lasted 5 min at each training session, for a total of 20 min. The therapist recorded the completion of daily training tasks using a notebook. The control groups were administered rewards in the same way as the corresponding experimental groups.

### 2.4. Outcome measures

The primary and secondary outcomes were assessed at baseline, after the intervention, and 3 months after the intervention.

#### 2.4.1. Primary outcome

The Chinese-Wechsler Young Children scale (C-WYCSI; Gong Yaoxian, 1992), revised by Gong in 1986 and based on WPPSI, is an individually administered intelligence test that measures the intelligence level of children with IDD, to generate FSIQ, verbal IQ (VIQ), and performance IQ (PIQ) scores. Moreover, the subtests are linked to many cognitive functions, such as learning ability, attention, memory, and visual perception. The verbal scale includes information, vocabulary, arithmetic, similarities, and comprehension tasks. The performance scale includes animal egg, picture completion, maze, geometric design, and block design or visual analysis tasks. The C-WYCSI is applied to children aged 4–6.5 years, with an internal consistency level of significance > 0.7. A cutoff value of 70 distinguishes normal from IDD.

#### 2.4.2. Secondary outcomes

The Vineland Adaptive Behavior Scale-Chinese Version (VABS-C; Yao and Gong, 1991) is a well-validated multidimensional instrument that measures adaptive behavior skills and overall severity of IDD. It consists of three main scales—daily life skills, communication, and socialization—tested on eight subscales: sensorimotor development (six items); self-care skills (10 items); speech and language development (nine items); personal orientation (10 items); social responsibility (nine items); space–time orientation (four items); labor skills (seven items); and economic activities (four items). The daily life skills scale comprises subscales for sensorimotor development, self-care skills, labor skills, and economic activities; the communication scale, speech and language development and space–time orientation; and the socialization scale, personal orientation and social responsibility.

The VABS-C is suitable for children aged 3–12 years and has a level of internal consistency significance > 0.8. A total adaptive behavior score (ADQ) and three sub-area scores (daily living skills, communication, and socialization) provide an overview of adaptive functioning in children with IDD. Higher scores indicate better adaptive behavior skills. A cutoff value of 70 distinguishes normal from adaptive behavior deficits.

### 2.5. Statistical analyses

A blinded statistician performed all data analyses using SPSS statistics V.24.0 (IBM SPSS Statistics for Windows, IBM Corp, Armonk, NY, United States). The statistical significance level was taken as 0.05. Baseline demographic and clinical characteristics data (i.e., child’s sex, primary caregiver, and disorder) were assessed using independent samples t-tests and chi-square tests. This study used an intention-to-treat analysis and imputed missing data by multiple imputations of the SPSS statistics software. For each intellectual function and adaptive functioning, a 2 (group) × 3 (time) linear mixed model (LMM) for repeated measures was used to test the effects of the intervention. This study specified time (baseline vs. post-training vs. follow-up) and group (experimental vs. control) as fixed effects in this model. In contrast, between-participant mean differences (i.e., the intercepts) were modeled as random effects. We followed significant interaction effects using *post hoc* tests with paired-samples t-tests. In addition, Holm–Bonferroni corrections were applied to the results of the interaction effects on the three cognitive abilities measured.

Pearson’s correlation analyses were then performed between the intellectual function and adaptive functioning changes that showed significant time × group interaction effects. We also investigated whether VIQ mediated the group intervention effect on changes in adaptive performance for significant intervention effects on the changes in adaptive functioning that correlated with the change in verbal intelligence due to the intervention. We performed mediation analyses using the PROCESS macro (Hayes, 2013) implemented in SPSS, with the group as the independent variable, VIQ as the mediator variable, and changes in adaptive functioning after the intervention as the dependent variable. In the mediation models, any mediating effect of VIQ was reflected as the indirect effect. The PROCESS macro was based on ordinary least-squares regression and adopted a nonparametric bootstrapping procedure (5,000 times), which gave rise to a bias-corrected confidence interval (CI) for effect size inference (Shrout and Bolger, 2002). A significant effect as *p* < 0.05 indicated if zero was not included within the 95% CI (Preacher and Hayes, 2008).

## 3. Results

**Figure 2:**
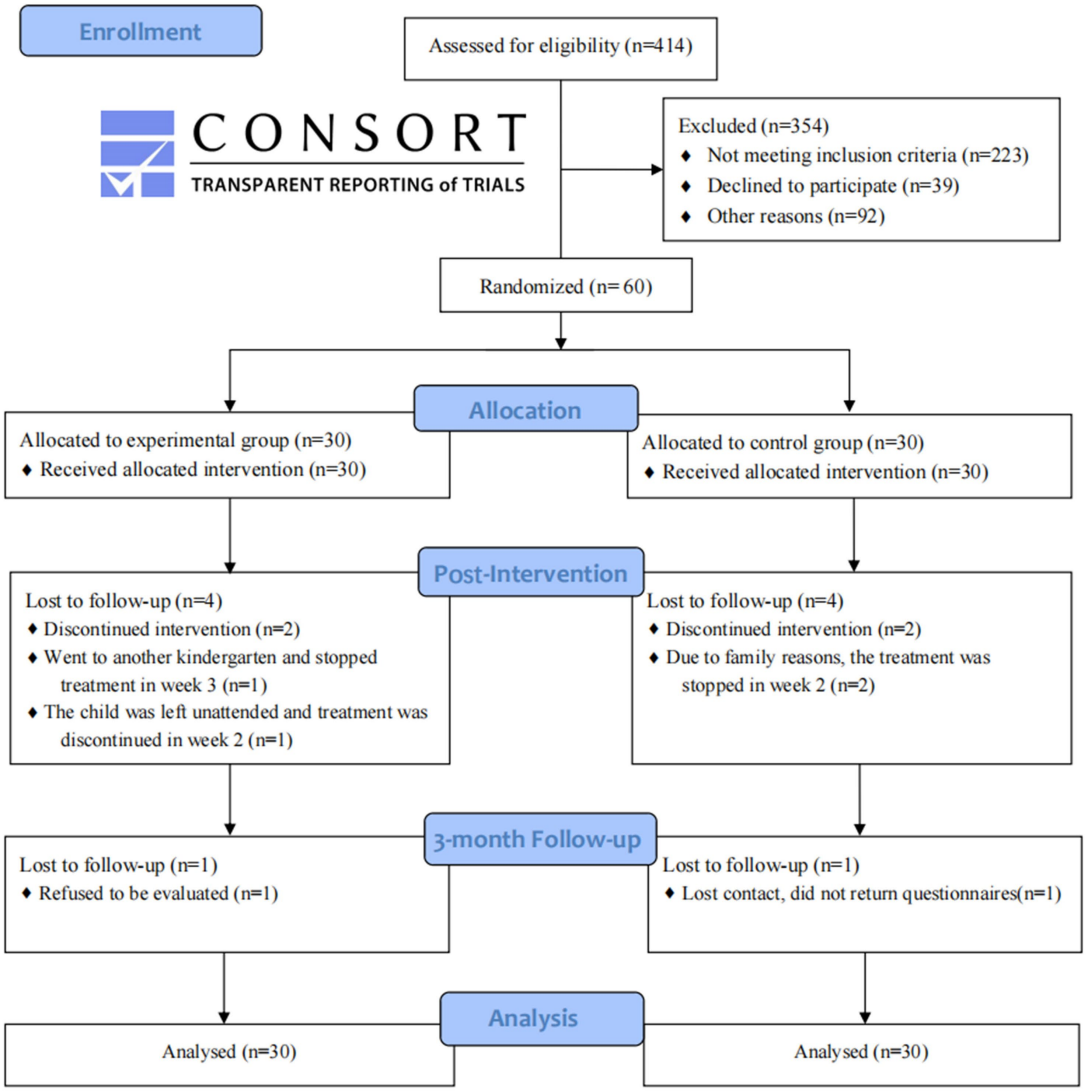
CCT study protocol and participant flow according to Consolidated Standards of Reporting Trials (CONSORT) guidelines.

A total of 414 children were screened, 304 of whom met the exclusion criteria. Therefore, 60 children with mild IDD were finally included in this study (Figure 2).

### 3.1. Baseline demographic and clinical characteristics

Thirty children in the intervention group and 30 in the control group were analyzed. The baseline characteristics of the intervention and control groups are given in Table 1. The mean chronological age of the intervention group was 5.13 ± 0.74 months, with 24 boys and six girls, and the mean chronological age of the control group was 5.03 ± 0.89 months, with 26 boys and four girls. There was no significant difference between the experimental group and the control group in terms of chronological age, sex, primary caregivers, and parent marital status (*p* > 0.05).

**Table 1:** Baseline demographic and clinical characteristics.

Characteristic	Experimental group (*n* = 30)	Control group (*n* = 30)	*t*/*χ*^2^-value	*p*-value
**Demographic**				
Child’s chronological age (years)^&^	5.13 ± 0.74	5.03 ± 0.89	−0.39	0.694
Males/females (*n*)^#^	24/6	26/4	0.48	0.488
**Primary caregiver of child**				
Parental care/relative care (*n*)^*^	23/7	28/2	NA	0.145
**Parental marital status**				
Single-parent family/two-parent family (*n*)^*^	27/3	29/1	NA	0.612

& Data are expressed as mean ± SD or frequency.

# Pearson Chi-Square test.

* Fisher’s Exact test. Experimental group, multi-domain computerized cognitive training; Control group, multi-domain traditional cognitive training.

### 3.2. Intervention effects on IQ

#### 3.2.1. FSIQ

**Figure 3:**
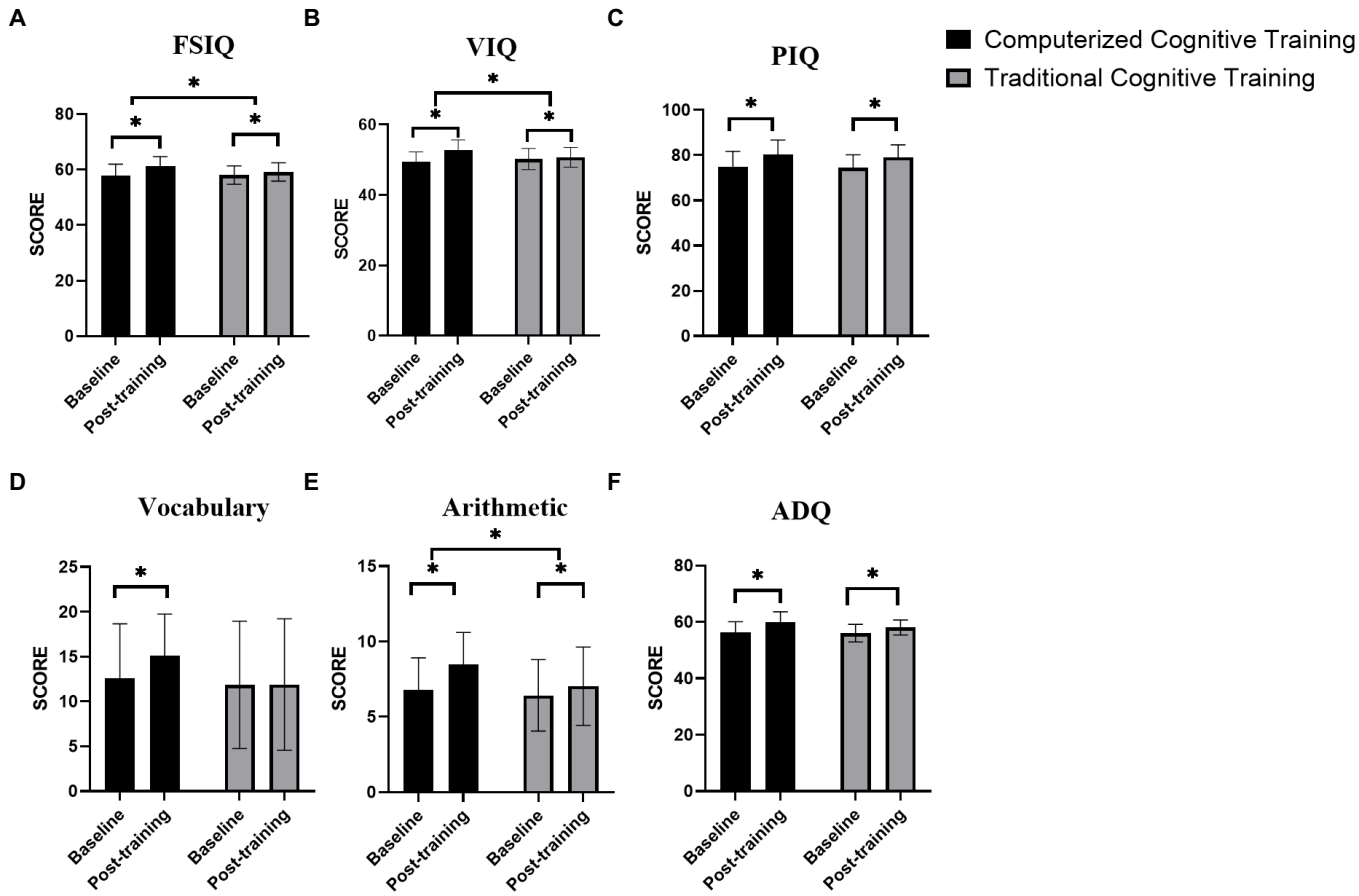
Within-group comparisons of **(A)** FSIQ, **(B)** VIQ, **(C)** PIQ, **(D)** arithmetic, **(E)** vocabulary, and **(F)** adaptive functioning scores for the baseline and post-training assessment occasions. Horizontal lines indicate significant *post hoc* comparisons with paired t-tests (^*^p ≤ 0.05, two-tailed). Significant group × time interaction effects were found in FSIQ, VIQ, and arithmetic.

The results of the LMM analysis showed that the overall FSIQ scores significantly increased after training (main effect of time: *F*[58] = 272.06, *p <* 0.01), and the time × group interaction effect was also significant (*F*[58] = 31.97, *p <* 0.01). Specifically, there were significant increases in scores on the FSIQ for the CCT group (*z*[29] = −4.69, *p* < 0.01) and the control group (*z*[29] = −3.71, *p <* 0.01) after training (Table 2; Figure 3).

**Table 2:** Summary of results of mixed linear model analyses on score of different clinical outcome measures of participants in the multi-domain computerized cognitive training group (Experimental group) and multi-domain traditional cognitive training group (Control group) for the baseline, post-training, and 3-month follow-up assessment occasions.

Outcome	Experimental group (*n* = 30)			Control group (*n* = 30)			Liner mixed model						Within-group comparisons (post-pre)			
	Baseline	Post-training	Follow-up	Baseline	Post-training	Follow-up	Time		Group		Group*time		Experimental group		Control group	
	Mean (SD)	Mean (SD)	Mean (SD)	Mean (SD)	Mean (SD)	Mean (SD)	t	p	t	p	t	p	t	p	t	P
FSIQ	57.83 (4.11)	61.11 (3.58)	63.63 (3.19)	58.03 (3.31)	59.14 (3.30)	61.26 (2.65)	272.06	<0.01	2.23	0.14	31.97	<0.01^*^	−4.69	<0.01^*^	−3.71	<0.01^*^
VIQ	49.50 (2.73)	52.79 (2.86)	55.26 (2.47)	50.23 (3.00)	50.68 (2.78)	53.33 (2.73)	175.01	<0.01	2.25	0.14	33.83	<0.01^*^	−10.59	<0.01^*^	−2.84	<0.01^*^
Arithmetic task	3.00 (0.79)	4.54 (0.84)	5.30 (0.67)	2.87 (0.82)	3.29 (0.81)	4.00 (0.62)	163.48	<0.01	26.28	<0.01	29.94	<0.01^*^	−4.57	<0.01^*^	−3.46	<0.01^*^
Vocabulary task	3.27 (0.91)	4.04 (0.92)	4.74 (0.81)	3.33 (0.88)	3.36 (0.91)	3.93 (0.83)	63.98	<0.01	5.56	0.02	17.30	<0.01^*^	−6.01	<0.01^*^	−1.00	0.32
PIQ	74.97 (6.73)	80.25 (6.42)	79.52 (5.77)	74.33 (5.88)	79.07 (5.48)	77.37 (4.79)	183.19	<0.01	0.63	0.43	1.06	0.35	−16.59	<0.01^*^	−9.83	<0.01^*^
Animal egg task	8.73 (1.72)	9.43 (1.79)	9.52 (1.76)	8.63 (1.50)	9.07 (1.65)	9.07 (1.69)	73.60	<0.01	0.56	0.46	6.14	<0.01^*^	−4.47	<0.01^*^	−3.31	<0.01^*^
Picture completion task	6.13 (1.04)	6.21 (1.03)	6.96 (0.94)	6.10 (1.03)	6.29 (1.01)	6.81 (0.88)	65.59	<0.01	0.01	0.93	0.70	0.50	−1.41	0.16	−2.00	0.05^*^
Mazes task	5.17 (1.23)	5.18 (1.25)	5.22 (1.25)	5.30 (1.02)	5.29 (0.98)	5.30 (0.99)	0.97	0.38	0.14	0.71	0.98	0.38	−1.00	0.31	0.00	1.00
Geometric design task	6.17 (1.51)	6.54 (1.53)	6.56 (1.55)	5.77 (1.43)	6.11 (1.40)	6.26 (1.29)	6.60	<0.01	0.89	0.35	0.31	0.73	−2.89	<0.01^*^	−1.52	0.13
Block design task	6.97 (1.43)	7.46 (1.26)	8.07 (1.07)	6.93 (1.72)	7.21 (1.62)	7.37 (1.52)	27.27	<0.01	0.61	0.44	5.05	0.01^*^	−3.00	<0.01^*^	−2.14	0.03^*^
ADQ	56.30 (3.81)	59.96 (3.66)	62.96 (2.98)	56.07 (3.10)	58.04 (2.66)	61.11 (2.53)	676.95	<0.01	2.22	0.14	28.05	<0.01^*^	−4.71	<0.01^*^	−4.68	<0.01^*^
Daily living skills	28.40 (2.70)	28.57 (2.10)	29.04 (2.03)	28.33 (1.90)	28.43 (1.83)	28.52 (2.05)	1.07	0.35	0.23	0.64	0.29	0.75	−1.51	0.13	−0.45	0.66
Communication	32.20 (4.68)	34.32 (3.99)	35.07 (3.25)	31.07 (3.70)	32.00 (3.08)	32.74 (2.67)	43.11	<0.01	3.23	0.08	10.86	<0.01^*^	−4.23	<0.01^*^	−2.45	0.01^*^
Socialization	12.40 (4.01)	16.11 (3.72)	19.37 (3.67)	12.67 (3.91)	15.36 (2.83)	18.89 (3.00)	840.38	<0.01	0.02	0.90	4.35	0.02^*^	−5.51	<0.01^*^	−4.78	<0.01^*^

FSIQ, Full-scale Intelligence Quotient; VIQ, Verbal Intelligence Quotient; PIQ, Performance Intelligence Quotient; ADQ, Adaptive Developmental Quotients. For this measure, the total sample size was 60 (experimental group: 30, control group: 30), and the results of the linear mixed model comparisons were obtained after controlling for age, sex, and child’s clinical disorder.

* *p* < 0.05.

#### 3.2.2. VIQ

The results of the LMM analysis showed that the overall VIQ scores significantly increased after training (main effect of time: *F*[58] = 175.01, *p <* 0. 01), and the time × group interaction effect was also significant (*F*[58] = 33.83, *p <* 0.01). Specifically, there were significant increases in scores on the VIQ for the CCT group (*z*[29] = −10.59, *p* < 0.01) and the MCT group (*z*[29] = −2.84, *p <* 0.01) after training (Table 2; Figure 3).

#### 3.2.3. Arithmetic and vocabulary of VIQ

Significant group × time effects were revealed on the arithmetic (*F*[58] = 29.94, *p <* 0.01) and vocabulary (*F*[58] = 17.30, *p <* 0.01) scores. Both the CCT (*z* = −4.57, *p <* 0.01) and MCT (*z* = −3.46, *p* < 0.01) groups showed significant increases in arithmetic after completing the training programs. Significant increases were observed in vocabulary in the experimental group from the baseline to post-training (*z* = −6.01, *p <* 0.01). In contrast, participants in the control group did not show significant differences between these two assessments (*z* = 1.00, *p* = 0.32; Table 2; Figure 3).

#### 3.2.4. PIQ

The results of the LMM analysis showed that the overall PIQ scores significantly increased after training (main effect of time: *F*[58] = 183.19, *p <* 0.01), whereas no significant increase was observed for the time × group interaction effect (*F*[58] = 1.06, *p* = 0.351). Specifically, there were significant increases in scores on the PIQ for the CCT group (*z*[29] = −16.59, *p* < 0.01) and the MCT group (*z*[29] = −9.83, *p <* 0.01) after training (Table 2; Figure 3).

#### 3.2.5. Animal egg and block design/visual analysis of PIQ

Significant group × time effects were revealed on the scores of the animal egg (*F*[58] = 6.143, *p* = 0.003) and block design/visual analysis (*F*[58] = 5.046, *p* = 0.008). Significant increases in scores on the animal egg and block design/visual analysis were observed in both the CCT (*z* = −4.47, *p <* 0.01; *z* = −3.00, *p <* 0.01, respectively) and MCT groups (*z* = −3.31, *p <* 0.01; *z* = −2.14, *p* = 0.03, respectively; Table 2).

### 3.3. Intervention effects on adaptive functioning

For the ADQ, the LMM analysis showed that the main effects of time (*F*[58] = 676.95, *p <* 0.01) and time × group interaction effect (*F*[58] = 28.05, *p <* 0.01) were significant. *Post-hoc* tests showed that the ADQ of the participants in the experimental (*z*[29] = −4.71, *p* < 0.01) and control groups (*z*[29] = −4.68, *p* < 0.01) increased significantly after training compared to the baseline (Table 2; Figure 3).

#### 3.3.1. Communication and socialization of ADQ

Significant group × time effects were revealed for communication (*F*[58] = 10.86, *p <* 0.01) and socialization (*F*[58] = 4.351, *p* = 0.015) scores. Significant increases in scores for communication and socialization were observed in both the CCT (*z* = −4.23, *p <* 0.01; *z* = −5.51, *p <* 0.01, respectively) and MCT groups (*z* = −2.45, *p <* 0.01; *z* = −4.78, *p <* 0.01, respectively; Table 2).

### 3.4. Change of VIQ mediates the intervention effect on ADQ

**Figure 4:**
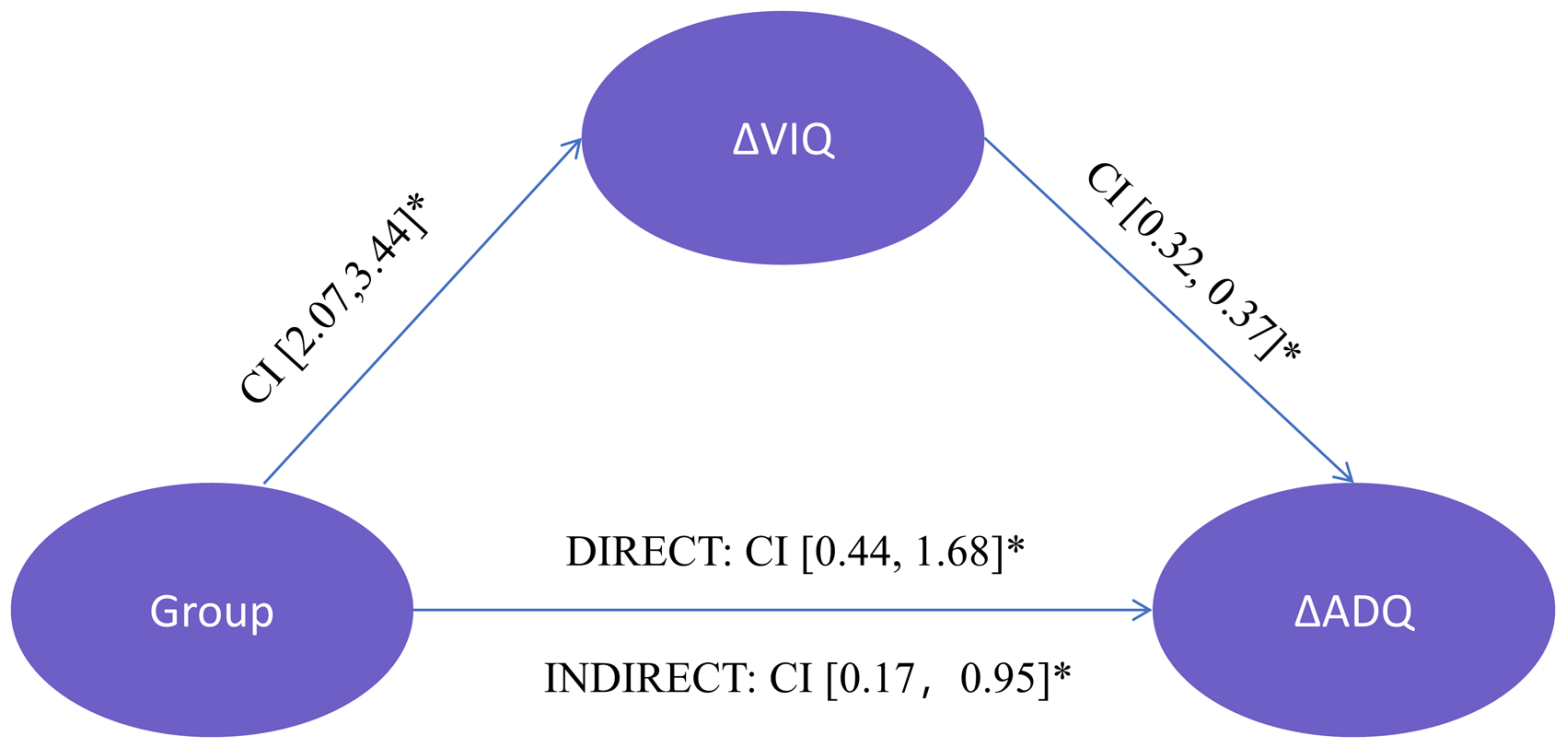
The VIQ change (ΔVIQ) due to the intervention mediates the group’s effect on ADQ change (ΔADQ). Group was the independent variable (experimental = 1, control = 0), ΔVIQ was the mediator variable, and ΔADQ was the dependent variable. Beside each arrow is the bootstrapping confidence interval (CI) of each effect. ^*^p < 0.05.

**Figure 5:**
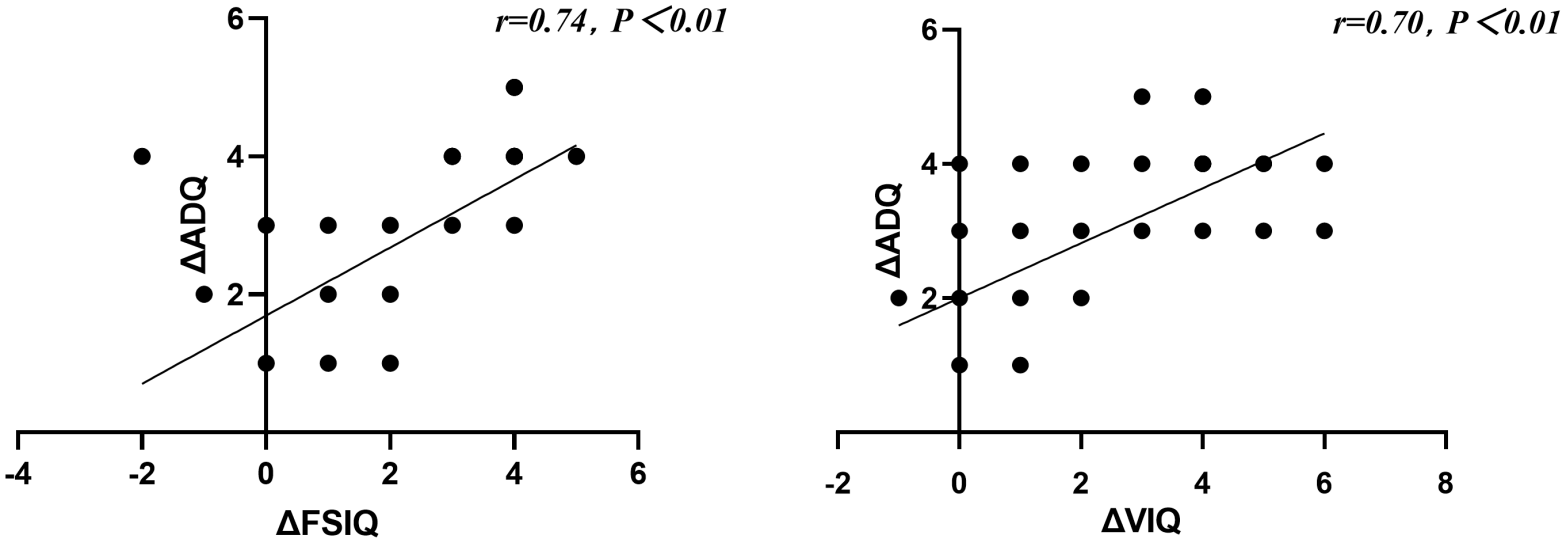
Correlation analyses performed between the changes of the intelligent function and adaptive functioning that show significant time × group interaction effects.

The change in FSIQ and VIQ (ΔFSIQ and ΔVIQ) were found to be significantly correlated with the change of the ADQ performance after training (ΔADQ; r[60] = 0.74, *p <* 0.01, r[60] = 0.70, *p <* 0.01, respectively) across the groups. Thus, we explored whether the ΔVIQ following the intervention mediated the group effect on ΔADQ. We conducted mediation analyses with the group as the independent variable, ΔVIQ as the mediator variable, and ΔADQ as the dependent variable. The analyses revealed that the mediating effect of ΔVIQ was significant and positive (bootstrapping CI: [0.17, 0.95]), and the direct effect was also significant (bootstrapping CI: [0.44, 1.68]). Specifically, the group (experimental group coded as 1, control group coded as 0) had a positive effect on the ΔVIQ (bootstrapping CI: [2.07, 3.44]). This was consistent with the finding that the experimental group showed greater improvement of VIQ following the intervention which, in turn, had a positive effect on ΔADQ performance (bootstrapping CI: [0.32, 0.37]). Therefore, IDD children with a greater improvement of VIQ following the multi-domain CCT showed a more significant increase in ADQ, reflecting better adaptive functioning (Figure 4). Correlation analyses between the changes of the intelligence function (r_s_ = 0.74, *p* < 0.01) and adaptive functioning (r_s_ = 0.70, *p* < 0.01) showed significant time × group interaction effects (Figure 5).

## 4. Discussion

We found that a 5-week multi-domain CCT can significantly enhance the intellectual performance and adaptive functioning of children with IDD. The improved intellectual function is positively connected with improved adaptive functioning. Moreover, the increase of verbal intelligence mediated the training-induced increase in adaptive functioning.

CCT outperforms traditional cognitive training in the FSIQ, VIQ, and animal egg and block design/visual analysis in the sub-items of PIQ. The improvement of intellectual function and cognitive function in children with IDD induced by CCT is consistent with previous studies. Van der Molen et al. (2010) showed that 5 weeks of computerized memory training increased intellect, short-term linguistic memory, and story recall. Kirk et al. (2016) observed that selective attention was enhanced after 5 weeks of computerized attention training in IDD children aged 4–11 years, and the positive impact was still substantial after 3 months. These single-domain CCT studies found a better effect on domain-specific improvement. However, although these studies found that CCT training had a good effect on cognitive impairment in a single domain, its generalization effect to other cognitive domains was limited (Bennett et al., 2013; de Vries et al., 2015).

In this study, we used a multi-domain CCT. Some research reveals that integrating different cognitive functions into a successful cognitive training program can compensate for the restriction and instability of CCT training impact in a single cognitive field (Cortese et al., 2015). The animal egg task is related to hand-eye coordination, attention, and memory. The block design/visual analysis task assesses visual-motor coordination and abstract conceptualization, indicating that multi-domain CCT has certain advantages in improving the attention, memory, and visual-motor coordination of children with IDD. Previous studies have shown that multi-domain cognitive training can increase functional integration within and between different brain networks (Cao et al., 2016) and contribute to the functional remodeling of brain neuroplasticity (Gates and Sachdev, 2014). After training, the children showed enhanced neural activity in the orbitofrontal, superior frontal, middle temporal and inferior frontal cortices in the inhibition paradigm (Hoekzema et al., 2010). As a result, multi-domain CCT may support cognitive ability improvement in different dimensions of IDD children, resulting in superposition and “migration” effects, which can improve overall cognitive function and intelligence.

Several studies also showed that CCT can improve the academic achievement of children with IDD (Söderqvist et al., 2012; Dunning et al., 2013; Kirk et al., 2015; Kirk H. E. et al., 2017). This is consistent with the results of this study, showing that the improved PIQ scores are closely associated with learning ability in children with IDD in post-training. In addition, we noted a decrease in PIQ scores during the follow-up period, which may have decreased due to the reduced cognitive training they received. This suggests that cognitive interventions can help improve PIQ in children with IDD, but longer-term ongoing interventions are needed to ensure stable and sustained improvement in children’s cognitive abilities. Future studies should conduct longer-term interventions and observations to further clarify this point.

We also found that multi-domain CCT brings about a significant improvement in ADQ, communication, and social factor indicators, and FSIQ was positively correlated with ADQ. These findings are consistent with prior CCT research. Computerized attention training increased behavioral control in children with fetal alcohol syndrome disorders, according to Coles et al. (2018), which improved adaptive functioning in daily life. Ko et al. (2020) discovered that computerized attention training could positively affect children’s adaptive behavior with cognitive impairment; Lanfranchi et al. (2017) noticed that computerized memory training could significantly improve the working memory of children with Down syndrome, and this ability after training can be transferred to self-care in daily life. Several studies (Lightbody and Reiss, 2009; Camprodon-Rosanas et al., 2019; Uddin, 2021; Chen et al., 2022) found that activating brain areas associated with cognitive function improves behavioral performance. The neural functions behind specific cognitions influence adaptive behavior in real-time (Lightbody and Reiss, 2009). Therefore, improvements in comprehensive cognitive performance can be transferred to adaptive functioning, resulting in improved adaptive behavior in children.

We found that the 5-week training improved the arithmetic and vocabulary tasks of IDD children. In terms of arithmetic task, the experimental group increased 1.54 points, while the control group increased 0.77 points. In terms of vocabulary task, only the experimental group had significant differences compared with the pre-intervention. Kroesbergen et al. (2014) studied 51 children with low working memory ability and found that 4 weeks of working memory training can promote the improvement of early computing ability. Yang et al. (2017) also found that 3-week computerized working memory training can significantly improve children’s reading fluency. The theory of internal cognitive load shows that cognitive ability is closely related to academic skills, and that academic skills competency requires the use of a variety of cognitive resources (Tabbers et al., 2004). In this study, the multidisciplinary CCT improved vocabulary and arithmetic skills after training and during the follow-up period. This continuous effect may relate to the multidisciplinary cognitive training that improves overall cognitive ability, so that IDD children can perform better in academic skills.

We found that CCT can indirectly promote the improvement of children’s adaptive functioning by improving verbal intelligence. This improvement in adaptive behavior may manifest in communication and socialization. Verbal intelligence is essential for children to communicate and socialize (Soorya et al., 2015; Hodge et al., 2021; Lyons, 2021). Multi-domain CCT promotes the recovery of language intelligence due to the rich audio-visual interaction (Takacs et al., 2015; Jaschke et al., 2018) and daily life scenes (Helo et al., 2021) in the training module. This also suggests that promoting language intelligence may be an essential aspect to improve the adaptive behavior of IDD children, which requires attention in the future training of IDD children.

There are several limitations in the current study. First, this study is not a double-blind randomized controlled clinical trial. We used blinding methods for the outcome evaluators and statisticians to minimize interference with the experimental results. Second, the sample size was small due to resource limitations and strict inclusion criteria. Therefore, future studies should conduct multi-center randomized controlled clinical trials with larger sample sizes to further validate the effects. Third, most children with IDD included in this study had mild intellectual disabilities. Future studies could consider examining the effects of CCT on moderate or severe intellectual disability children with IDD. Finally, this study focused on analyzing the effects of CCT on cognition and related functions in children with IDD after 5 weeks of training. There may be a learning effect for repeated assessments within a short period of time, which limits the extrapolation of CCT efficacy. To better elucidate the long-term effects of CCT on intellectual and adaptive functioning in children with IDD, future studies could be conducted for longer periods of time, and techniques such as functional magnetic resonance imaging and near-infrared brain imaging could be used to explore the neural mechanisms and provide more physiological evidence for the training effects.

Our findings suggest that multi-domain CCT can improve the results of intelligence and adaptive functioning tests in children with IDD. In addition, the improvement of adaptive functioning induced by cognitive training may be due to the enhancement of verbal IQ.

## Data availability statement

The raw data supporting the conclusions of this article will be made available by the authors, without undue reservation.

## Ethics statement

The studies involving human participants were reviewed and approved by Fujian University of Traditional Chinese Medicine Affiliated Rehabilitation Hospital Ethics Board for the project. Written informed consent to participate in this study was provided by the participants’ legal guardian/next of kin.

## Author contributions

JW and JP: conceptualization, methodology, investigation, writing—original draft, and project administration. ZL and HD: resources, writing—original draft, and writing—review and editing. ZH, YH, JT, and LC: methodology, supervision, and writing—review and editing. JH: methodology, resources, writing—review and editing, supervision, and funding acquisition. All authors contributed to the article and approved the submitted version.

## Funding

This study was supported by the Guiding Project of Fujian Provincial Department of Science and Technology, China (2022Y0038).

## Conflict of interest

The authors declare that the research was conducted in the absence of any commercial or financial relationships that could be construed as a potential conflict of interest.

## Publisher’s note

All claims expressed in this article are solely those of the authors and do not necessarily represent those of their affiliated organizations, or those of the publisher, the editors and the reviewers. Any product that may be evaluated in this article, or claim that may be made by its manufacturer, is not guaranteed or endorsed by the publisher.

## Abbreviations

ADQ: Adaptive developmental quotient
CCT: Computerized cognitive training
CI: Confidence interval
IDD: Intellectual developmental disorder
ITT: Intention-to-treat
LMM: Linear mixed model
RCT: Randomized controlled trial
